# Stories From Black Women in Iowa About Reproductive Health Care Experiences, Self‐Advocacy, and Recommendations for Change

**DOI:** 10.1111/hex.70609

**Published:** 2026-03-06

**Authors:** Melissa Lehan. Mackin, Nicole Loew, Stephanie W. Edmonds, Ann Weltin, Lynette Cooper, Lastascia Coleman

**Affiliations:** ^1^ University of New Mexico, College of Nursing Albuquerque USA; ^2^ University of Iowa, College of Nursing Iowa USA; ^3^ Abbott Northwestern Hospital part of Allina Heath Minneapolis USA; ^4^ University of Iowa, College of Medicine Iowa USA

**Keywords:** Black women, cultural humility, iatrophobia, implicit bias, Iowa, patient‐centred care, patient‐provider relationship, qualitative research, racism, reproductive health

## Abstract

**Research Question:**

What are Black women's experiences when seeking reproductive health care in Iowa?

**Design:**

Narrative‐style qualitative research.

**Methods:**

Adult Black women in Iowa were recruited for phone interviews. Data was analysed using within and across case methodology.

**Results:**

Clinical experiences included suboptimal healthcare, a lack of information, and being treated differently because they were Black. Consequences included not returning to the provider, mistrust of providers and health systems, but also greater self‐advocacy.

**Conclusion:**

The findings from this study are illustrative of the realities that Black women in Iowa faced when seeking reproductive healthcare.

**Implications for the Health Professionals and Patient Care:**

Women in this study voiced a need for health professionals to actively listen to people under their care, demonstrate genuine human concern, and adopt person‐centred and shared decision‐making models for care delivery. Recommended system changes include a need to further diversify the workforce, improve training for the care of persons of colour, and develop better clinical tools that consider different skin and body types.

**Patient and Public Involvement:**

Members of the public were involved in the study design and interpretation of findings. Their contributions included providing feedback on research proposal drafts and attending research team meetings to ensure that the research itself did not contribute to racism. Community members with the shared identity of research participants also provided an interpretive lens for the data, crucial for the understanding of white research team members who have not experienced racism.

## What Does This Paper Contribute to the Wider Global Clinical Community?

- Clinical experiences for women in this study included suboptimal healthcare, a lack of information, and being treated differently because they were Black and illustrated discrimination that occurs in the context of the patient provider relationship.
- Recommended system changes include a need to further diversity the workforce, improve training for the care of persons of colour, and develop better clinical tools that consider different skin and body types.

## 1 Introduction

Structural inequities that exist for Black women in the healthcare system and contribute to poor health outcomes have been well‐documented and include racialized and gendered disadvantage, in addition to disproportionate access to resources, and health payor sources. (see as examples [1, 2, 3]). Additional reports have identified disadvantage specific to the reproductive health domain resulting in increased morbidity and mortality, poor pain management, and lack of relevant clinical guidelines and decision‐making norms for Black women (see as examples [4, 5, 6]). The resulting racial health disparities are well‐documented [7, 8] with evidence accruing for how the relationship between racism and chronic stress contributes to poor health [9, 10, 11, 12].

The impacts of structural racism greatly contribute to the current reproductive health landscape for Black women (see Table 1 for definitions of racism). In the US, Black women die from reproductive‐related conditions up to four times the rate of white women, with maternal mortality rates higher than any other developed country [7, 8, 14]. Black women are also disproportionately affected by HIV [15, 16, 17], infertility [18, 19], gonorrhoea, syphilis, and chlamydia [17].

**Table 1:** Definitions of racism and implicit bias.

Racism [13]	A system of oppression that assigns value to persons based on the social construction of physical characteristics intended to define race and ethnicity.
Institutional racism [13]	Prejudice and discrimination perpetrated by organisations or governments that impose practices that have negative outcomes on access to health services and subsequently affect the quality of care.
Implicit Bias (American Psychological Association, 2024)	A negative attitude, of which one is not consciously aware, against a specific social group.

In addition to structural racism, racism experienced in the context of the patient provider relationship and clinical encounter can also impact health outcomes. Although the body of literature focusing on racism in this context is small, previous research has identified that racism in clinical interactions results in dismissal of the patient voice, paternalistic decision making, and discriminatory treatment decisions [20, 21]. These interactions impact trust and future health‐seeking behaviour and can result in iatrophobia, or ‘fear of the healer’ [20, 21, 22]. Iatrophobia is especially relevant for Black women due to the history of medical racism they have experienced [23, 24, 25, 26, 27].

While a complete discussion of the history of medical racism for Black women is beyond the scope of this paper, a brief discussion provides important context for the shared community memories of exploitation Black women have experienced at the hands of medical providers. Specific to the focus on reproductive healthcare, four examples of historical mistreatment are provided here as illustrations.

Example 1. During enslavement, the value of a Black woman was determined by her ability to produce more enslaved people [25]. Reproduction for Black women was often a result of rape or forced breeding practices by her enslaver for the purposes of financial gain. The lack of control over reproductive health was compounded by medical experimentation on enslaved Black women in the name of advancing science [27].

Example 2. J. Marion Sims, ‘The Father of Gynaecologist’, is commemorated for perfecting surgical repair of vesicovaginal fistulas. However, he achieved this status through surgeries on numerous enslaved Black women without their consent or use of anaesthesia [27]. Paradoxically, even after abolition and well into the 20th century, Jim Crow laws prevented Black women from accessing the medical care they had assisted to refine [27, 28].

Example 3. In the 1970s, the eugenics movement specifically targeted Black and Indigenous women with forced sterilisations during non‐related surgeries [29]. e.g., Black women often woke up from abdominal surgeries to find that a hysterectomy had also been performed. This happened so frequently that these procedures were sardonically named ‘Mississippi appendectomies’ [30].

Example 4. More recently in the 1990s, Norplant, a long‐term contraceptive, was an implement of government programmes to curb reproduction in poorer communities and promoted primarily to Black women [31, 32, 33, 34]. Programmes were implemented in places such as inner‐city Baltimore, where school‐based programmes promoted its use in a large population of Black adolescents [35] without parent consent [25]. Additional problems arose when these women wanted removal which required a visit to a health provider [36, 37]. Women provided Norplant through government programmes were often unable to afford or access a provider for removal, essentially preventing their reproductive autonomy. While not the direct result of actions by health providers, providers were part of a system which made them proxies in allowing the narrative of excessive Black reproduction to continue, perpetuating attempts to exert reproductive control over Black women. Uncovering how structural forces and individual decision‐making converge is central to understanding use of medical services.

While many people express shock and dismay when learning of these events, to Black women, they are well known and shared community knowledge. Iatrophobia is an ever‐present obstacle between Black women and health providers [23, 26, 27, 38] and manifests as mistrust and lack of confidence in medical advice and care or avoidance of healthcare altogether. This consequence demonstrates additional impacts of racism on health.

Racism underpins differences in health outcomes that persist even when other variables such as education and socio‐economic status are controlled [28, 39, 40, 41, 42]. Specific to reproductive health for Black women, racism is at least partially responsible for low rates of cervical cancer screenings and mammograms [43], high rates of maternal mortality [44], and lack of adherence to hypertension [45] and HIV treatment [46].

Volpe et al. [47] examined state and provider‐level racism in a large study population of non‐Hispanic Black and white people (*N* = 21,030; *n* = 2,110 Black people). In the US healthcare landscape, more state‐level racism increased the likelihood that white people had better access to care, more time spent with the provider, and better quality of care. In this same study, racial discrimination against Black persons by providers was associated with 80% lower odds of having a provider explain care, 77% lower odds of answering questions, and 68% lower odds of having enough time with the provider than when compared to white persons.

Racism is more likely to be experienced in geographic areas where the racial majority is white and without ethnic/racial diversity [48]. These communities maintain implicit biases because they remain unchallenged, as may be the case with Iowa. Iowa is one of few states where > 90% of the population and > 98% of healthcare providers are white [49].

Iowa is considered a rural state with about two‐thirds of Iowans living away from metropolitan cities that tend to have greater racial diversity [50]. Rural areas also have a shortage of healthcare providers and 77% fewer public clinics that offer contraceptives compared to urban areas [51]. These conditions likely contribute to why Black women in Iowa disproportionately experience poorer reproductive health outcomes. When compared to other racial categories, Black women in Iowa are least likely to initiate prenatal care and most likely to have a pregnancy before age 20, experience a pregnancy‐related complication, and deliver a low birth weight or pre‐term infant [52].

While research examining system‐level and provider level impacts of racism in the establishes a strong foundation, few of these studies have sought perspectives directly from Black women. Three studies were identified that used the voices of Black women to describe their reproductive health experiences. Two of these studies were conducted in urban settings and all focused on a specific demographic segment of Black women. F. M. Howell [53] examined middle‐class Black women's experience of gendered racism when seeking reproductive health care in New York City. The researcher presented findings that reported impacts of intersections of race, class, and how experiences can only be understood in the context of social, economic, historical, and political forces. Adebayo, Parcell, Mkandawire‐Valhmu, and Olukotun [54] studied pregnant Black women in Milwaukee, WI. Of specific focus was on provider communication practices that were interpreted as insensitive, unfair, and a dismissal of concerns. Logan, Daley, Vamos, Louis‐Jacques, and Marhefka [55] interviewed young, Black women (age 19‐29) about their experiences during family planning visits in various U.S. geographies. All studies supported the importance of the patient provider relationship, trust, and effective communication.

The research reported here sought to contribute to this growing body of literature while giving voice to Black women in a number of ways. Given Black women's reproductive health disparities and the lack of racial diversity in Iowa, there was a critical need to examine the reproductive health experiences of Black women in this rural geography. Additionally, there was a need to better understand *how* clinical experiences are attributed to health and health behaviour. Therefore, the purpose of this study was to describe the experiences of Black women and answer the research question, *What are Black women's experiences when seeking reproductive health care in Iowa and what is the impact on future care seeking?*

### 1.1 Statement of Researcher Positionality

The research team was composed of a mix of researchers, community members, and clinicians. We were mindful that our personal identities and experiences could influence our approach and interpretations. For this reason, the authors provide readers with the following information about our backgrounds. MLM is a non‐Hispanic, white, cisgender nurse researcher with an interest in women's health, health equity, and has extensive qualitative research experience. NL is a non‐Hispanic, white, cisgender PhD prepared nurse with qualitative research experience. NL is a proponent of anti‐racist health care reform and has sought a state‐level political office. SE is also a non‐Hispanic, white, cisgender PhD prepared nurse with qualitative research experience. SE conducts clinical research within a health system and actively works toward anti‐racist health reform. AW is a non‐Hispanic, white, cisgender nurse practitioner and midwife who has a history of serving Black women immigrants. MLM, NL, SE, and AW do not have the lived experience of racism. L. Cooper and L. Coleman are the founders of the BWMHC. L. Cooper is a Black, cisgender nurse practitioner with a passion for anti‐racism and health equity. L. Coleman is a sought‐after public speaker about needed change in the health care environment and how the clinical environment should serve Black women. L. Coleman is a Black, cisgender nurse midwife currently seeking her PhD and directs the midwifery programme at her institution. She is also a widely sought‐after speaker to talk about the state of health for Black women and maternal mortality and the need for greater racial congruence of providers in the clinical setting. Both L. Coleman and L. Cooper have personally experienced racism in the reproductive health setting.

## 2 Methods

A qualitative design using phone interviews was employed to give voice to Black women's experiences seeking and receiving reproductive health care in Iowa. This qualitative research is guided by the principles of community‐engaged research [56, 57]. Community‐engaged research is an academic and community partnership that promotes bi‐directional relationships that can inform all phases of the research process and is commonly used in research focusing on health disparities [56]. Black Women's Maternal Health Collective (BWMHC), a grassroots group of Black health service providers and leaders, partnered with researchers for this study. The BWMHC was co‐founded by two of the authors on this paper (L. Coleman and L. Cooper). The group provides a space for Black women to share pregnancy and birth experiences in a community of love and support of other Black women [58]. The partnership of researchers with BWMHC facilitated mutual learning, trust building, and promoted a shared vision of health improvement. Members of the BWMHC played an important role of assisting the whole of the research team to understand their positionality within this research and the larger context of health care. Specifically, community partners were an integral part of the research team. Their roles included refinement of the research question, review and revision the interview guide, proposal of the story telling methodology, interpretation of the data, and drafting portions of this manuscript.

To participate, participants had to be: (1) An adult Black woman, (2) Reside in the state of Iowa; and (3) Be able to speak and read English. Women were primarily recruited via social media that included community Facebook pages, Twitter (now X), and also by word of mouth. Interested persons were screened for inclusion and then contacted to schedule the interview.

A semi‐structured interview guide was developed to explore how Black women recount their experiences accessing reproductive health care and was situated within storytelling research methodology [59]. The choice to use this methodology was purposeful for several reasons. Qualitative storytelling approaches to data collection are particularly relevant and a preferred method for cultural groups, like Black Americans, that have historically used storytelling as a form of communication. It has the additional benefit of reducing the negative connotations of an ‘interview’ associated with traditional research language [59]. This methodology has been used previously to communicate experiences of racism [60] and to gain a deeper understanding of contexts where health decision making occurs [61]. Table 2 provides the primary interview questions used for this research.

**Table 2:** Primary questions included in the interview guide.

*Can you tell me a story about your most memorable experience when you received reproductive health care?*
*Have you sought health care to get birth control? Can you tell me a story about your experience seeking birth control from a provider?*
*Can you tell me a story about healthcare experiences that influenced your reproductive health outcomes or choices?*
*Have you ever been afraid to seek health care? Can you tell me a story about a time where you were afraid to seek health care or your feelings prevented you from seeking health care?*
*Can you tell me a story about how you might change what happened or what was said when seeking or receiving health care to make the interaction more positive?*
*Have you ever felt that treated differently than other women when seeking reproductive care? Can you tell me a story about an experience Why do you think you were treated differently?*
*The goal of this research study is to better understand how experiences of discrimination impact health care and health seeking. Is there anything else you think we should know as we try to make positive changes in how you or other people experience health care?*

As a baseline measure of how women in this study perceived racism, the Perceptions of Racism Scale was used in this study [62]. The tool examines the affective feelings about racism, the experience of racist actions, and how racism is internalised. The tool has performed well in two study samples reporting Cronbach's alpha scores of 0.88 and 0.91, respectively [62]. Responses were assigned a number from 1 to 4 and summed with larger scores indicating higher perceptions of racism. For the current study, women either completed this measure using an electronic survey prior to the interview or it was administered during the interview. Due to the small sample size, only descriptive statistics were used to interpret this data.

A total of 12 interviews were conducted over a period of 4 months in early 2023. All interviews were conducted by two consultants, both Black women, nurses, and members of the BWMHC. Both nurses had training and experience conducting qualitative interviews. The use of racially congruent interviewers strengthened the credibility of the study by potentially increasing the rapport with the women they interviewed. Interviews were conducted by phone and recorded and lasted 45–90 min. Interview recordings were professionally transcribed with all identifiable information removed and names replaced by pseudonyms assigned to each participant. Pseudonyms allowed humanisation of stories and were carefully chosen not to reinforce common stereotypes about Black women. Transcribed data in the form of text documents were managed with the use of an excel file that documented the staged process of within and across case analysis. As each interview was completed, it was holistically coded and themed by two members of the research team and which allowed for the identification of case‐level patterning. Case level interpretations were then brought to the whole team for confirmation using discussions to refine and revise initial findings. Next, three members of the research team examined case‐level profiles to identify similarities, differences, recurring processes, or contradictions. This approach allowed for interpretation of both the ‘general context’ and the context of ‘each individual's account of experience.’

The number of interviews was determined to be sufficient when no new themes were derived from additional interviews (*n* = 2). Theoretical validity was established by returning the themes identified during across case analysis to individual cases for refinement and to search for competing or conflicting interpretations. To further enhance trustworthiness of the findings, all coding decisions and themes were presented to the larger research team and discussed, resulting in mutual agreement, with the final codes and themes presented in this paper. The research strategy and involvement of community partners played an integral role in providing alternative explanations or interpretations of the data to ensure that findings were grounded in participants' realities, ultimately enhancing the credibility, dependability, and confirmability of the data.

Ethical Considerations. This study was approved by the University of Iowa, Institutional Review Board (Study number 202104667) in 2021. Participants were monetarily compensated for their time. The Consolidated Criteria for Reporting Qualitative Research (COREQ) was followed for reporting findings for this study.

## 3 Results

Twelve Black women living in Iowa participated in this study (A summary of background characteristics of the sample is provided in Table 3). Ages of women in this study ranged from 20 to 53 years. When asked about the city/town in which they live, most participants (*n* = 10; 83.3%) were from three cities (populations 70–200 thousand people) and considered more urban in comparison to the general rurality of Iowa, given that 904 of 947 Iowa cities have less than 10,000 people [63]. Almost all women (*n *= 11; 91.7%) reported access to primary care services and practiced wellness care in addition to seeking care when they were ill. The majority of the sample (*n* = 9; 75.0%) had previously experienced pregnancy and childbirth. Of the 10 participants that completed the *Perceptions of Racism Scale*, scores ranged from 51 to 71 (highest possible score 80) and mean score of 62.8, suggesting that women in this study's perceived racism was moderate to high (Cronbach's alpha = 0.85).

**Table 3:** Characteristics of study participants.

Participant characteristics (*N* = 12)^a^	
Age	
Range	20–53 years
Mean	37.7 years
Score on perceptions of racism scale (*N* = 10, highest possible score = 80)	
Range	51–71
Mean	62.8
Length of time in Iowa	
Range	1–53 years
Mean	18.3 years

	*N* (%)
Access to primary care provider	11 (91.7)
Access to wellness services	11 (91.7)
History of pregnancy/childbirth	9 (75%)

^a^ Sample size *N* = 12 unless otherwise specified.

Due to the complexity of the topics discussed during the interview three central themes were identified to organise the results each followed by subthemes. The three central themes include: (1) *Clinical Experiences*, 2) *Outcomes of Clinical Experiences*, and (3) *Needed Practice and System Changes*. We provide a brief description of the central themes followed by expounded explanations of subthemes. Subthemes are presented using the women's words as labels supported by direct quotations. This approach was done to maintain the story element of how information was shared and to elevate the voices of the women who wanted to share their care experiences. Names in parentheses are pseudonyms assigned by the research team along with the age of the participant.

### 3.1 Core Theme 1: Clinical Experiences

*Clinical Experiences* describes the challenges that Black women faced when seeking reproductive healthcare. All women in this study described circumstances where they received suboptimal care or were treated differently. Suboptimal care came in subtle forms such as lack of information, being presented with limited options (e.g. oral birth control pills only), and getting what they felt was poorly reasoned advice for their complaints (e.g. lose weight to stop vaginal discharge). Some women reported more signs of poor treatment (e.g. refusing to stop vaginal exam) or being refused treatment (e.g. pain relief). Almost all women in the study had a story about feeling physically or emotionally invisible, not being listened to, and/or feeling underinformed or uninformed. Three subthemes included; ‘Nobody told me’, ‘They just didn't see me’, and ‘Being Black has a lot to do with it.’

### 3.2 Subtheme 1: ‘Nobody Told Me’

Almost all women reported that they felt that they did not receive enough information, especially surrounding how their body works and available care options. Sometimes things providers said were not clear, such as ‘My primary physician had me go to an OBGYN. I was like, ‘What? First, what is O‐B‐G‐Y‐N?’ (Tayri, 36). Several other women included in their stories how not having information preemptively made future complications surprising and scary. ‘They said my heart muscle got weak after I had my child. I was put on a heart meds…I was never told that my heart could get weaker…I ended up having a heart transplant’ (Breanna, 53). ‘There wasn't a relationship between me and the doctor. You go in, get your pills, keep it moving. I don't think that I was ever given any information that I can remember in retrospect about how to take care of my body [due to side effects]’ (Dianna, 53). Other women reported lack of information in regards to only being offered limited options. This seemed particularly salient when women sought birth control. ‘They just asked me was I healthy… [Then] he was like, ‘Well, I think Depo would be a really good option for you. It's low maintenance. [Here you go!]' We didn't go over the side effects’ (Cecilia, 23). ‘My doctor really pushed one specific birth control. I was interested in other ones, but they really were pushing the one that they prescribed me…Most likely I think it was because I was as a young Black woman, I think that my doctor felt that she knew what was best for me, and that I wasn't able to make the decision myself’ (Iris, 20).

### 3.3 Subtheme 2: ‘They Just Didn't See Me’

Not being seen, being invisible, and as an extension, not feeling like providers were listening, was a prominent subtheme across interviews (*n* = 10; 83.3%). Invisibility was described as physical, ‘I stood there for three or 4 min, and those young women just kept talking like they just didn't see me there, so I just sat’ (Dianna, 53). For Dianna this resulted in feeling disrespected and distrustful of health workers.

Not being seen also occurred in the form of being physically visible but having concerns ignored or dismissed. ‘While I was in the hospital, I started my period. I was having some very, very painful cramps, and I was bleeding an abnormally constant amount. I was in the hospital for a total of, maybe, 5 days. The days that I was there, I had been complaining the entire time about the issue, and I wasn't attended to until the day I left. A doctor come in and give me a vaginal exam with a speculum. It was my first exam…that [experience] was scary for me and I felt like I wasn't really being heard or understood’ (Iris, 20). ‘When I was in pain while I was pregnant, they tried to say that ‘oh, it's just the pregnancy. That's why you are in pain, but I would tell them, I know the difference between pregnancy pain and my lupus pain because this is not my first child. This is my second child’ (Evette, 25). For one woman in this study, not being listened to resulted in sexual violence and retriggering of a past trauma. Cecilia (23) shared, ‘When I was younger, I was sexually assaulted, so when I'm getting a pap smear or exam, I'm really uncomfortable. I'm usually in tears, it's really difficult…I went to a primary care provider for an exam, I asked her to stop and take a break. She was like, ‘No, I'm almost finished. We'll get it done quickly so then you're not uncomfortable.' I was like, ‘I'm already uncomfortable’.

### 3.4 Subtheme 3: ‘Being Black Has a Lot to Do With It’

The majority (*n* = 10; 83.3%), but not all women in this study directly attributed poor treatment in the healthcare setting to their Black identity. As one example, Patrice (24) associated her Black identity and negative experiences to implicit biases. ‘I definitely know that being Black has a lot to do with it…It's not the first or last time I'll experience lack of compassion when working with a healthcare provider… [health providers'] assume the strength of Black women… it's a tough diagnosis or it might be life‐changing, but she'll survive. She's used to that’ [*sarcastically*]. Josie (34) summarised her experiences by saying, ‘I never thought I would experience micro‐aggressions and flat‐out racism, so it's been interesting.’ For Kiki (31), differential treatment was related to her Black identity but also her age and being an unpartnered mother. ‘I might feel like they're treating me different on my end because I'm the same Black girl that's in here delivering kids and their father's not on the side. I might feel like they're treating me different because all these other women that they see come in are with their spouse, and I'm always alone, maybe with my mom’. Dianna's (53) experiences prompted this advice for providers, ‘[Ask yourself] was this my undercover racism coming to the forefront?’

### 3.5 Central Theme 2: Outcomes of Clinical Experiences

*Outcomes of Clinical Experiences* describes how women's interactions with clinicians impacted health outcomes and future health‐seeking behaviours. Lack of trust was the most common outcome but consequences were not always described as negative. Within this theme, three subthemes were identified, ‘Trust is not my experience’, ‘it's very important to advocate for yourself’, and ‘I've been afraid to seek healthcare.’

### 3.6 Subtheme 1: ‘Trust Is Not My Experience’

Women's stories reflected a lack of trust in both the health system and with individual providers from whom they sought care. Distrust stemmed from historical medical racism and personal experiences and sometimes resulted in changing providers or not returning for care. ‘As a Black woman, I don't say I have complete trust in the medical field. Don't know that I ever will’ (Patrice, 24). Josie (34) shared, ‘I speak to some of my friends…They are just so trusting of their provider and [their providers] ask all the right questions and provide all the solutions and the options. [My friends] feel empowered to make good decisions about their own personal health. I was just like, wow. [Having trust] is not what my experience was or how I felt’.

### 3.7 Subtheme 2: ‘It's Very Important to Advocate for Yourself’

Stronger self‐advocacy was an outcome of negative clinical experiences expressed by two women in the study. ‘I had become an advocate to speak up for myself, to ask questions, to develop relationship with my doctors. With the doctors that I have now, I can look them in the eye and I can talk to them. Going through all that has made me know that it's very important to advocate for yourself, and just don't let them sell you anything’ (Breanna, 53). Tayri's (36) experiences prompted her to have a frank discussion with her provider about the importance of her Black identity, ‘I spoke to [my provider] about being a Black woman, and I was like, ‘And you are a white woman, there's a difference, and I need you to focus on me being a Black woman and the healthcare that is needed for me.’ Kiki (31) lamented that her lack of self‐advocacy may have contributed to her negative care experiences. ‘Maybe I should've spoken up more. Maybe in different times that I've been to see a health provider, I could've spoken up and said different things. Even with the birth control when I tried it the first time, and it had all those different effects on me that I wasn't aware of, I could've spoken up about that, and maybe asked more questions.’

### 3.8 Subtheme 3: ‘I've Been Afraid to Seek Healthcare’

About half of the women in this study expressed fear of health providers and the system, although only two reported that their iatrophobia prevented them from seeking appropriate health care for their complaints. ‘[Due to past experiences] I've been afraid to seek healthcare. [One time] I was feeling really sick, but there weren't any symptoms that you could see or identify immediately…I got so I was experiencing bad pain. I was having headaches and sinus pain and ear pain as well as queasiness and an eye infection…I just felt like I wasn't going to be taken seriously or nothing was going to be done. [I ended up with hearing loss in both ears]’ (Olani, 37). Josie (34) aptly summarised the consequence of iatrophobia, ‘The fear that exists, and the impact that it has on their relationship with future providers and the sense of mistrust in the healthcare system and not knowing whether or not you're prioritised, if your health is prioritised’.

### 3.9 Core Theme 3: Needed Change

*Needed change* describes actions and changes that women in this study suggested be made by individual clinicians and to the healthcare system. Women in this study expressed specific strategies they felt were necessary to improve the care that Black women receive. At the provider level three subthemes included: ‘Start actually listening’, ‘genuinely concerned,’ and ‘Giving me the choice to make’. At the health system level, three additional subthemes included: ‘I did not see one Black doctor,’ ‘How do you not know what to do?’, and ‘That stinky chart is not made for Black women’.

### 3.10 Needed Provider Changes

Women had specific suggestions of how the patient provider relationship could be improved. Recommendations for individual providers included active listening, exhibiting genuine concern, and mutual decision making.

### 3.11 Subtheme 1: ‘Start Actually Listening’

While some women in this study directly communicated a need for providers to be better listeners, ‘I would just say they need to start listening to people and actually listening’ (Cecilia, 23). Other women shared this sentiment by describing the impact of experiences where they felt providers had listened to them. ‘We had a real thorough conversation [about birth control]. That was the first conversation that I had where I felt like she was listening to me, and that was big. That's when I realised, I was like, ‘Okay. I can talk to you, and you will listen’ (Tayri, 36). ‘I suffered from endometriosis. It just was painful, but at the end [after we developed a relationship] I had doctors that would listen to me as best they could’ (Dianna, 53).

### 3.12 Subtheme 2: ‘She Was Genuinely Concerned’

Like the subtheme listening, some women were direct in their recommendation while others shared stories of the impact of when providers demonstrated genuine concern. When asked about how care could be improved, Dianna (53) responded **‘**Just that genuineness that you're concerned about me.’ Dianna also provided an example from her experience, ‘It was the relationship that I had with her – that she didn't see me just as another Black woman who's about to have her third child…She was genuinely concerned because I was like, ‘I got one kid in diapers. We can't do this again.’ Kiki (31) also discussed her satisfaction with her current provider who is white, because of the genuine concern Kiki perceives. ‘I feel like she's not against me. She's with me…her being my doctor, there's certain things that I could talk to her about that I can't talk to my family about, so I may say something, and me thinking I'm going to get another [negative] response, it's the opposite. I get nothing but good things from her. She lets me know. Just do this. Try this. If this doesn't work, do this. I just feel like she's just on my side.’

### 3.13 Subtheme 3: ‘Giving Me the Choice to Make’

Suggested improvements in the clinical setting also included women's wish to be empowered to make informed decisions in partnership with their providers. ‘With Black women and our reproductive health and historically how we've been treated with forced sterilisation and different things like that. I think that I just have the right to make a decision about my body’ (Dianna, 53). Dianna underscored her comments with an example of a positive health interaction, ‘I think being able to weigh the options of what's best for me and with my other health issues and then having those conversations with my husband as to what would fit best and what our concerns are as a couple. I appreciate her giving me information and just allowing me to take the information back and make those decisions with my husband.’ And another example, ‘I didn't want to get on birth control because it was going to mess up my body. She ended up talking me into doing what's best for me…She just basically went through and told me different information about the different birth controls that they have to offer and what they do. She didn't sugarcoat anything. She was so honest with me. The birth control that I actually did get on, she's on the same one, so she knows. She told me about her experience. She won with that one’ (Kiki, 31).

### 3.14 Needed Systems Level Change

Suggested system level changes went beyond the individual provider to extend more generally to the health care system. Challenges that need to be overcome include a more diverse workforce, increased education of health providers, better clinical tools, and larger societal level culture change.

### 3.15 Subtheme 4: ‘I Did Not See One Black Doctor, Nurse’

Black women in this study expressed a need for more Black providers that understand and share the lived experience of being a Black person. ‘I can't do anything about more Black people going into the healthcare field, but I wish they would because [Black people] are number one people for heart disease, diabetes, all the bad stuff’ (Breanna, 53). Breanna also shared, ‘My daughter had a baby. I did not see one Black doctor [in a large medical centre]. I didn't see one Black nurse. I really want all Black providers, and I have consistently looked for African American doctors. It's unfortunate because I cannot find them’ (Raina, 39). Olani (37) extended these wishes to not just providers but the whole health setting, ‘I would like to see more Black people around in the hospital, I want to see Black people greeting me when I come in, I want to hear Black music as I'm sitting in the waiting room, or Black magazines, and something that's just like, ‘You're welcome here too'.’

### 3.16 Subtheme 5: ‘How Do You Not Know What to Do?

A need for better cultural rigour and implicit bias training was the most often cited recommendation for the health arena. Some women described experiences that demonstrated a lack of provider training and knowledge in the care of Black people. Olani (37) reported ‘I went to see a dermatologist because I had a rash. She said, ‘I've never treated a Black person before, so what do you think should be the treatment?’ and I was like, ‘I don't know, I'm not a dermatologist.’ That experience is definitely in my mind whenever I go to any sort of doctor*—*my eye doctor, reproductive doctor*—*any doctors. ‘Have you even treated Black women before?’ and ‘Are you either going to lie to me and be like, ‘Yeah, this is what I think is going to happen,’ or*—*be honest, ‘I don't know what to do?’ but also, ‘How do you not know what to do?'‘. Other women expressed a need to address racism in healthcare. ‘You can't say, oh, yeah, people are racist, but not me. I had a nurse say, ‘I don't know a white supremacist.’ That, to me, reveals that you're just not educated on white supremacy…. People have unlearning and relearning to do’ (Patrice, 24). ‘…Diversity and cultural competency to me – once you decide that you want to become a medical professional, that should start at day one, not day two’ (Dianna, 53).

### 3.17 Subtheme 6: ‘That Stinky Chart Is Not Made for Black Women’

Two participants shared the need for better clinical tools and approaches that are based on Black bodies. ‘But you know you keep pulling' out that little stinky [BMI] chart. That stinky chart is not made for Black women. It's made for white women. It's not even an average’ (Dianna, 53). ‘I would definitely want to see just more acceptance of Black body types. It's triggering when I go in and, ‘Oh, we need the extra big arm cuff for your blood pressure,’ and stuff like that, it's like, ‘Why don't you all just have arm cuffs that fit different bodies in the hospital room, or gowns that fit? Those things I think would be respectful’ (Olani, 37).

## 4 Discussion

The findings from this study are illustrative of the realities that Black women in Iowa faced when seeking reproductive healthcare. The perspectives shared by these women portray clinical experiences that contribute to the understanding of the relationship Black women have with the current health system, the consequences of those experiences (e.g. iatrophobia, healthcare avoidance), and how history and personal experience might compound to create poorer health outcomes.

Several of the women in this study described instances of not having enough information to manage health concerns. Having health information that is understandable and complete is a crucial antecedent to making informed decisions about health. The reported lack of health information by the study participants is demonstrative of the health system‐wide problem of health literacy [64]. Almost 90% of adults in the U.S. have limited health literacy, with the highest prevalence of poor health literacy among marginalised and poor people. The failure to communicate information in a way that is easily comprehended by health consumers and avoids jargon, unjustly favours people who have higher education levels and higher health literacy, typically white people and Asian/Pacific Islanders [65, 66, 67]. Low health literacy is a known negative social determinant of health resulting in systemic disadvantage for Native, Hispanic, and Black people [68, 69].

It was disheartening to hear about the experiences that comprised Subtheme 2: ‘They just didn't see me’ and Subtheme 3: ‘Being Black has a lot to do with it’. There were numerous reports of women not being seen or heard or what they did say was dismissed. At first, this lack of visibility appeared juxtaposed to being visible as a Black woman. However, the stories as a whole underscore the perspective shared by several women in this study that Black women are seen as less human and have been stereotyped as able to endure higher levels of pain [70]. It is this normalised racism that can explain how Black women can be both invisible and discriminated against because of what providers see [54, 71].

This study's findings about lack of visibility and dismissal of concerns were not new, but reflective of previous research. Treder et al. [72] examined healthcare professional bias and discrimination experienced by Black women. Like reports in our study, Black women participants reported little shared decision‐making and experienced invalidation and dismissal of their health concerns. The resulting lack of trust in the provider catalysed the need to employ self‐protective actions such as overpreparing for appointments, enlisting advocates, and only seeking care when desperate. Like the research findings reported by Treder et al., women in our study felt a disconnect in the patient‐provider relationship which is antithetical to promoting health and wellness.

Within the stories of shared clinical experiences, for a few participants these were ‘lessons learned' and contributed to the ability to be better self‐advocates with their providers. While these perspectives represented an ideal outcome from negative experiences, it was much more common for women to feel frustration and remorse due to not receiving information about their conditions, options, or medication side effects. Although we are not claiming that individual providers acted in bad faith, their actions and attitudes often conveyed a provider‐centred framing, in which the options offered and information shared reflected what providers believed was best rather than fully supporting patient choice. While providers have the education and experience to make healthcare diagnoses and recommend best treatment, medical paternalism prevented opportunities for shared decision making and trust‐building. A paternalistic orientation compounds perspectives of racism and inequity by further eroding patient autonomy, satisfaction, treatment adherence, and patient engagement; all of which contribute to poorer health outcomes [73, 74, 75, 76, 77].

In this study, provider mistrust was caused or exacerbated by women's clinical experiences. This finding was not novel as the outcome of mistrust as a result of historic treatment and distressing healthcare experiences has been identified across several recent studies [72, 78, 79]. Treder et al. [72] examined the lived experience of racism for urban Black women specific to experiences in the reproductive health setting in a qualitative study (*N* = 21). Medical mistrust was only one of the consequences of racism experienced by women in their study. The authors' conclusion was a call for health providers to acknowledge the pervasive effects of racism and the stress endured by Black women interfacing with the health system. Mhaimeed et al. [79] in their scoping review concluded that medical mistrust is a barrier for Black people seeking health care that could be improved with the implementation of shared decision‐making models. Coward‐Murrell [78] conducted a small pilot study in North Carolina that surveyed Black adults (*N* = 25) to examine medical mistrust during a 30‐min patient encounter with a provider. When Black patients perceived that the provider demonstrated behaviours that would ease mistrust, two‐thirds of the sample reported feeling more comfortable with the information the clinician provided. The findings echo Mhaimeed et al.'s conclusions suggesting the potential for improved outcomes when medical mistrust is addressed in the patient‐provider clinical interaction and includes shared decision‐making.

Though our interviews did not ask about experiences of multiple marginalised identities, several women's stories exemplified how they attributed poor treatment in the health setting to being Black and a woman. Like other studies, women also cited weight, age, and disability as additional stigmatisations and attributions of the poor care they received. James‐Conterelli et al. [80] and Howell [53] discussed the potential impact of racism at the intersections of race and various identities such as being both ‘Black’ and ‘woman’. Impacts were compounded beyond what individuals with single identities experienced, making women with more intersectional identities more highly vulnerable to negative outcomes. Future research that includes interventions and actions to promote the health of those at most vulnerable is needed.

The stories shared by women in this study exemplify several implications for the need to improve clinical care. First, there is a need for more widespread adoption of shared decision‐making models that include patient‐ or person‐centred communication [81, 82]. Shared decision‐making includes asking patients culturally and medically appropriate questions relevant to individualising patient care [79, 80, 83]. Similar to patient‐centred care where the key is to build a partnership that supports decision‐making, patient‐centred communication elicits the patient's perspectives, understands that the patient has her own unique context, and ensures that treatments are concordant with the patient's values [81, 82]. Both shared decision making and person‐centred communication improve trust and safety, satisfaction with care, and assist to motivate for behaviour change [79, 80, 83, 84, 85, 86, 87, 88]. These strategies may be one way to reduce negative experiences such as those reported by participants in this study, such as feeling invisible, provider mistrust, not understanding medical jargon, and iatrophobia.

Many models for shared decision making that include patient or person‐centred communication exist and several have been adapted for specific patient populations (e.g. Dementia patients, asthma patients) in addition to those specific to reproductive health care. (see as examples [81, 82, 89, 90, 91, 92, 93, 94]).

Second, there is a need for more widespread adoption of trauma‐informed care approaches that can assist in reducing medical mistrust. This approach emphasises the impact of trauma on a person's behaviour, coping skills, and reframes the focus from ‘what is wrong with this person?’ to ‘what happened to this person?’ [95]. This response includes a heightened awareness of personal, historical, and racial trauma, demonstrates empathy, improves communication, and reduces the likelihood of triggering a trauma response in people under a provider's care [95].

Third, health literacy is a highly modifiable social determinant of health and improving the understanding of health information needs to be a disciplinary and organisational priority. While the women in this study discussed a lack of information from specific providers, the entire health system needs to be held accountable for enabling consumers to find, understand, and use information and services to make health‐related decisions [66]. System accountability and clear communication policies are mechanisms in which health organisations can improve dissemination of health information and reduce their contribution to systemic racism [96]. Future research is needed to identify and duplicate successful health literacy interventions, especially in populations that remain disadvantaged such as Black women.

Fourth, health providers need to be taught and practice cultural humility [97]. Cultural humility is different and moves beyond cultural competence which implies a clinician reaches full knowledge of a culture [80, 97]. Emphasising cultural humility shifts the role of expert health provider to the patient and requires the provider to engage in lifelong learning and self‐reflection and contributes to an ultimate goal of cultural rigour [98]. Cultural humility impacts patient care by improving patient outcomes, reducing misdiagnosis, decreasing healthcare costs, and moderating implicit biases. Due to this impact health institutions committed to anti‐racism should require all health professionals and students to receive rigorous training in cultural humility. Future research will need to continue to develop and adapt measures of cultural humility, cultural rigour, and implicit biases to allow agents to measure effectiveness of interventions, change, and growth.

Fifth, racial incongruence in health providers has been a long‐standing problem in the US healthcare landscape. As several women in this study articulated and was consistent with other research findings, people want care providers to know, understand, and relate to their personal identities. Racial congruence between a person and their provider contributes to better health outcomes. Institutional and organisational actions that can assist in diversifying the healthcare workforce include: purposefully directing employment advertising to target groups; diversifying interviewing and admission teams; providing extensive internship or training opportunities to support transition to practice; focusing on retention and engagement; facilitating leadership development opportunities; and cultivating a culture of inclusivity and belonging [99, 100].

Last, design of this study and the study findings suggest women's voiced need for improvements clinical tools that are relevant for Black women and do not promote additional stigmatisation or shame. Two women in this study specifically identified BMI as not representative or accurate for Black women's bodies. These women voiced what is widely acknowledged in scientific and lay literature that BMI is largely developed using white people's bodies as the norm and is socially constructed. Although this indicator may have limited usefulness at a population level, is not an accurate indicator of an individual's health or metabolic status and providers should avoid using BMI to classify individual's body mass or size [101, 102, 103]. Although not specific to Black women, the American Association of Clinical Endocrinology has issued a consensus statement aimed at improving care of persons with obesity including issues of body composition measurement, communication, and avoidance of weight bias and stigma that worsen outcomes [104].

In addition to the potential lack of fit of BMI to all body types, there are few empirically developed measures of racism. The Perceptions of Racism Scale [62] was used in this study to quantify the context of perceived racism. While the scale demonstrated evidence of reliability in this sample, all scores were in a narrow range and the small sample size prevented assessment of discrimination. In general, there are few measures of racism that exist that could identify a range of perceptions and be able to capture indicators of potential change in knowledge or attitudes. Additional research is needed to adapt or develop such a measure that health systems could ultimately use the tool to measure patient experiences and positive change in their institution.

While this study provides valuable insights into the experiences and perceptions of racism, several limitations must be acknowledged. First, Black women's experience in Iowa may be different than those experienced in other geographies. The study was conducted in a specific geographic and socio‐cultural context, which may influence the experiences and manifestations of racism reported by participants. However, in this study we also believe that Black women have a particular authority when speaking to the consequences of racial discordance within the health systems in Iowa. Furthermore, while Iowa provided a unique context for this research, many findings were supported by previous literature, suggesting transference of experiences to Black women not living in Iowa. Future research with a larger sample and from more diverse geographies would need to be conducted for confirmability. Second, it is possible that interpretations of the findings were biased by positionality related to some of the research teams' racial identities. Four of the co‐authors have white identities and do not have the experience of racism and may not have recognised an experience as discriminatory. However, in order to ameliorate this potential, the analysis team was composed of both white and Black researchers who discussed findings and potential alternate interpretations in a purposeful manner in order to enhance the credibility of the study findings. Last, we recognise that asking participants to discuss experiences of racism may be emotionally and psychologically challenging. While we attempted to be thoughtful about the way in which questions were asked and that all interviews were conducted by racially congruent members of the research team, this may have affected women's willingness to share openly. While we had no indication that our participants were withholding information, our plan with future research related to experiences of racism, is to more intentionally incorporate trauma‐informed approaches to minimise distress and promote candid sharing. We encourage other researchers in this area to take a similar approach.

## 5 Conclusion

Stories shared by the women in this research provided several examples of what and how racism or differential treatment was experienced in the reproductive health domain. While most of the women shared stories of poor treatment, it is also important to recognise stories that illustrated resilience and self‐advocacy. The study findings are a reminder that people who seek care from a provider are a whole person complete with historic and life experiences that come to the healthcare relationship with them. Many improvements are needed in clinical arena to improve trust, communication, and experiences. Future research should develop and validate measures of racism and inequity, while also identifying and translating effective interventions that improve patient–provider relationships and, in turn, reproductive and overall health outcomes.

## Author Contributions

All authors on this paper contributed to conceptualisation of the research, analysis and interpretation of data, draft writing and review for dissemination and funding acquisition.

## Ethics Statement

All research activities and team members were approved by the Institutional review board. Women who participated in this study affirmed consent to participate.

## Conflicts of Interest

The authors declare no conflicts of interest.

## Supporting information

COREQ_Checklist.

## Data Availability

The authors have nothing to report.
